# Approaches in Polymeric Nanoparticles for Vaginal Drug Delivery: A Review of the State of the Art

**DOI:** 10.3390/ijms19061549

**Published:** 2018-05-23

**Authors:** Gerardo Leyva-Gómez, Elizabeth Piñón-Segundo, Néstor Mendoza-Muñoz, María L. Zambrano-Zaragoza, Susana Mendoza-Elvira, David Quintanar-Guerrero

**Affiliations:** 1 Departamento de Farmacia, Facultad de Química, Universidad Nacional Autónoma de México, Ciudad de México 04510, Mexico; gerardoleyva@hotmail.com; 2 Laboratorio de Sistemas Farmacéuticos de Liberación Modificada, Universidad Nacional Autónoma de México, FES-Cuautitlán, Cuautitlán Izcalli 54714, Mexico; 3 Laboratorio de Farmacia, Facultad de Ciencias Químicas, Universidad de Colima, Colima 28400, Mexico; nmendoza0@ucol.mx; 4 Laboratorio de Procesos de Transformación y Tecnologías Emergentes en Alimentos, Universidad Nacional Autónoma de México FES-Cuautitlán, Cuautitlán Izcalli 54714, Mexico; luz.zambrano@unam.mx; 5 Laboratorio de Microbiología y Virología de las Enfermedades Respiratorias del Cerdo, Universidad Nacional Autónoma de México, FES-Cuautitlán, Cuautitlán Izcalli 54740, Mexico; seme@unam.mx; 6 Laboratorio de Posgrado en Tecnología Farmacéutica, Universidad Nacional Autónoma de México, FES-Cuautitlán, Cuautitlán Izcalli 54740, Mexico

**Keywords:** nanoparticles, nanocapsules, vaginal treatments, drug release, natural polymers, synthetic polymers

## Abstract

The vagina is a region of administration with a high contact surface to obtain local or systemic effects. This anatomical area represents special interest for government health systems for different sexually transmitted infections. However, the chemical changes of the vagina, as well as its abundant mucus in continuous exchange, act as a barrier and a challenge for the development of new drugs. For these purposes, the development of new pharmaceutical forms based on nanoparticles has been shown to offer various advantages, such as bioadhesion, easy penetration of the mucosa, and controlled release, in addition to decreasing the adverse effects of conventional pharmaceutical forms. In order to obtain nanoparticles for vaginal administration, the use of polymers of natural and synthetic origin including biodegradable and non-biodegradable systems have gained great interest both in nanospheres and in nanocapsules. The main aim of this review is to provide an overview of the development of nanotechnology for vaginal drug release, analyzing the different compositions of polymeric nanoparticles, and emphasizing new trends in each of the sections presented. At the end of this review, a section analyzes the properties of the vehicles employed for the administration of nanoparticles and discusses how to take advantage of the properties that they offer. This review aims to be a reference guide for new formulators interested in the vaginal route.

## 1. Introduction

The vagina has been used for a long time as a route for drug administration. The vaginal route offers many advantages over conventional oral administration, such as the avoidance of the gastrointestinal environment and the hepatic first-pass effect. Although it is traditionally used for local action, some drugs can permeate the vaginal mucosa and reach the bloodstream in sufficient concentrations to possess systemic effects. Likewise, for drugs that act locally in the female reproductive tract, topical vaginal application results in much higher drug concentrations and improved efficacy [1]. Currently, vaginal products are marketed in the form of creams, gels, tablets, capsules, ovules, foams, solutions, etc. These conventional pharmaceutical forms are associated with poor distribution and retention, mostly owing to the self-cleaning action of the vagina, in addition to their poor ability to modulate the fate of active compounds once they are released into the cervicovaginal mucus (CVM) [2,3]. The CVM is a heterogeneous and complex system of intercommunicating channels formed by mucin fibers and filled with aqueous fluid [4].

During the last two decades, nanotechnology-based delivery systems for topical vaginal therapy have gained increasing attention. To design an optimal delivery system for the vaginal site, it is important to fully understand the challenges of this unique site in order to improve the distribution, retention, pharmaceutical efficiency, and patient acceptability of the final formulations [1,2,4]. The use of polymeric nanoparticles and other nanocarriers for the vaginal administration of different pharmacologically active molecules and their capacities and limitations have been extensively reviewed by several authors in recent years [1,2,3,4,5,6]. The majority of the different nanosystems designed for vaginal use, either possessing intrinsic activity or utilized for drug delivery, are intended for the prophylaxis and treatment of many conditions affecting the female reproductive tract, including sexually transmitted diseases, fungal and bacterial infections, and cancer [1,2,3,4,6]. For drugs that act locally on the female reproductive tract, topical vaginal application results in a higher drug concentration and reduced adverse effects, as well as improved efficacy [1,4]. The highly folded epithelial surfaces of the vagina and normal vaginal clearance and discharge comprise some of the challenges for designing and optimizing nanosystems for administration to the vaginal site [1,4,6].

A major challenge for the design of polymeric nanoparticles is the study of their interactions with mucosal fluids/tissues, as well as biodistribution upon vaginal administration [2]. According to Vanić and Škalko-Basnet [6], nanosystems for mucosal drug delivery could be classified by their surface properties and their ability to be involved in mucoadhesion or mucopenetration in the following three major groups: (i) conventional (non-mucoadhesive); (ii) mucoadhesive nanosystems; and (iii) mucus-penetrating nanosystems, e.g. with polyethylene glycol (PEG).

This article reviews the use of different types of polymeric nanoparticles designed for vaginal administration. Polymeric nanoparticles, either of natural or of synthetic origin, are included. Some of these were employed as single delivery systems, whereas others are included in vehicles delivery systems. It is noteworthy that, for this review, only solid nanoparticles based on polymers (nanospheres and nanocapsules) are considered, while systems formed by other associations of polymers or lipids are not included.

## 2. Brief Description of the Anatomy of the Vagina

The vagina is a distensible tubular, fibromuscular organ that is approximately 9 cm long, extending from the cervix (lower part of the uterus) to the vestibule of the external genitalia, being positioned between the urinary bladder and the rectum. The walls of the vagina are covered by a mucosal tissue that forms a series of transverse folds denominated rugae (more prominent in the lower third of the vagina), thus increasing the available surface for drug absorption. The vaginal tissue is composed of four distinct layers: stratified squamous epithelium, lamina propria, fibromuscular layer, and tunica adventitia; the former two are usually referred to as vaginal mucosa. Functions of the vagina include receiving the erect penis and semen during coitus and ejaculation, and serving as a passageway for a newborn from the uterus and menses to outside the body [7].

Despite being commonly referred to as a mucosa, the normal vagina lacks glands; thus, the mucin content of the vaginal fluid has a cervical source. Additionally, other fluids, originating in the uterine cavity or in higher (from and through the tubes) or lower structures (vestibular glands), as well as from transudation through the vaginal walls and epithelial cells, can be found in the vaginal content [3]. The amount and composition of the vaginal fluid change throughout the menstrual cycle and this fluid is influenced by age, vaginal practices (such as douching), sexual stimulation, and intercourse, as well as by pathological conditions such as vaginal infections. For example, women of reproductive age produce fluid at a rate of 3–4 g/4 h, while in postmenopausal women, this production decreases to one half of the fluid volume [3,4]. The acidic pH of the vagina (ranging from 3.8–4.5) is considered a protective factor against pathogen proliferation, and it is suitable for *Lactobacillus* spp. growth; therefore, it is simultaneously a mechanism and the product of healthy vaginal-flora balance. Lactobacilli compete with other microbes for nutrients and secrete factors, such as lactic acid, that render the environment inhospitable to other bacteria and pathogens. Vaginal pH is an indicator of possible infections caused by microbial pathogens [1,3,4].

With this panorama of biological challenges, nanotechnology-based delivery systems are a suitable tool to improve distribution, retention, and pharmaceutical efficiency.

## 3. Definitions and Classification of Nanoparticles

The term “pharmaceutical nanotechnology” comprises an emerging multidisciplinary branch in this field, which is currently experiencing exponential growth, with products already on the market. In fact, nanotechnology is one of the key technologies of the 21st century [8]. It has important implications in the development of novel procedures and systems for disease diagnostics and therapeutics. Pharmaceuticals based on nanotechnology are constantly proposed in the literature and several have intellectual protection. These systems offer several advantages regarding conventional non-nanoengineered products. In general, these improved characteristics are explained by their tiny particle size (≤1 µm) and molecular behavior [9,10]. These advantages can be summarized in the following aspects: (a) decreased fed/fasted variability; (b) decreased patient-to-patient variability; (c) enhanced solubility; (d) increased oral bioavailability; (e) increased rate of dissolution; (f) increased surface area; (g) a lesser dose required; (h) a more rapid onset of therapeutic action; (i) increased drug-targeting ability; and (j) increased patient compliance. One main challenge in therapeutics is delivering the drug to the desirable site, including to mucosal membranes such as the vagina. Thus, conventional dosage forms exhibit poor biodistribution, limited effectiveness, undesirable effects, chemical degradation, clearance, and lack of selectivity. Nanosystems are solving several issues related to drug delivery such as prolonged circulation, improved drug localization, enhanced drug efficacy, and, in general, novel ways for spatial and temporal delivery [9,11,12,13]. Terms such as nanopharmaceuticals or nanostructured materials have been employed to describe these systems, which include micelles, carbon nanotubes, polymersomes, polymeric, metallic and lipid nanoparticles, nanocapsules, nanogels, nanofibers, dendrimers, brush polymers, quantum dots, nanocomposites, etc. Polymeric nanoparticles are the nanosystem most studied and reported in the pharmaceutical industry. These nanocarriers have demonstrated drug-solubility enhancement, protection against rapid degradation, and enhanced drug concentration in target tissues; therefore, lower doses of drug are required to transport drugs to the site-of-action (cell or tissue) via passive/active targeting. Attractive aspects for the vaginal route include that fact that polymeric nanoparticles are submicron-sized colloidal particles produced by mechanical or chemical means, in which a therapeutic agent can be dissolved in their dispersion, encapsulated within their polymeric matrix, adsorbed onto the surface, or chemically attached. In terms of size, an interval between 10 and 1000 nm, more typically 50–600 nm, is considered pharmaceutically acceptable (1 nm = 10^−9^ m). Polymeric nanoparticles include both nanospheres and nanocapsules. The difference between these two forms lies in their morphology and body architecture. Nanospheres are formed by a dense polymeric matrix, whereas nanocapsules are reservoir or vesicular systems composed of an oil core surrounded by a thin polymer shell [14,15,16].

Natural or synthetic polymers can be utilized to prepare polymeric nanoparticles. Although natural polymers such as proteins (i.e., collagen, albumin, gelatin, legumin, vivilin, etc.) and polysaccharides (i.e., alginate, agarose, starch, dextran, hyaluronic acid, alginate, chitosan, etc.) are biodegradable in vivo, these are used to a lesser degree because of their problems in preparation processes, the toxic ingredients involved, and the uncertainty of the source, the purity of the macromolecule, and potential antigenicity. Similar drawbacks have been reported for nanoparticles prepared by polymerization reactions due to the byproducts generated, which can be non-biocompatibles or, in fact, toxic. Therefore, synthetic pre-formed polymers are preferable in pharmaceutical systems. They must be generally recognized as safe (GRAS), particularly those that are biocompatible and resorbable under natural conditions. Choice of the polymer depends on the properties of the system, such as therapeutic application of the formulation, the targeted drug-release profile, biocompatibility, etc. Namely, for the vaginal route, polymers able to meet to the different conditions of the cavity, such as pH changes, mucoadhesivity, ease of mucous penetration, stability in vaginal fluids, changes in mucosal gels, etc., are preferred. The majority of nanoparticle- preparation-processes in this review consider two steps: (a) the formation of an emulsion (oil-in-water); and (b) the precipitation/gelation of the polymer into nanoparticles by the presence of a non-solvent medium. It is important to point out that stabilizers play an important role in avoiding the formation of large aggregates; their use needs to consider the administration route. For vaginal administration, several stabilizers can be used, and their selection requires consideration of their relationship with the mucosa. In fact, the development of polymeric nanoparticles for therapeutic purposes has evolved in terms of considering the properties of the layer coating as follows: from first-generation nanoparticles, which are captured in the liver by the reticulo endothelial system (RES); into the second-generation stealth nanoparticles, which are coated with water-soluble polymers, especially PEG, and which increase systemic circulation and passive targeting; passing on to third-generation nanoparticles with targeting moiety, where the layer coating is formed by a ligand specific for receptors expressed in tumor cells; and lastly to the ideal nanoparticle archetype (fourth-generation), represented by multifunctional nanoparticles, which combine diagnostic agents with therapeutic agents and a reporter of therapeutic efficacy in the same system [17,18,19,20,21].

**Figure 1:**
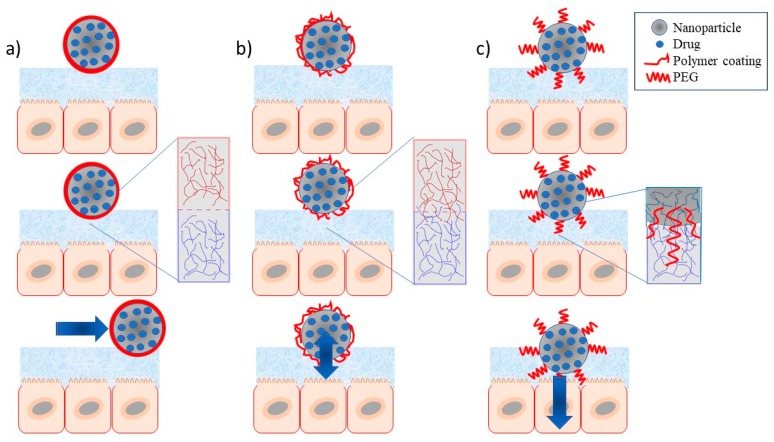
Schematic representation of the nanoparticles with different coating surfaces and their ability to interact with the mucus layer: (**a**) non-mucoadhesives (non-interacting layer); (**b**) mucoadhesives (polymer layer); and (**c**) mucus-penetrating nanosystems (PEG layer).

Recently, researchers working with mucosal drug-delivery systems (i.e., ocular, nasal, oromucosal-bucal, sublingual, gingival, pulmonary, rectal, gastrointestinal, and vaginal sites) have proposed a new classification that considers the surface properties of the nanosystems and their capacity to interact with the mucus gel and, more specifically, with *O*-glycosylated macromolecules, the main component of the mucus responsible for mucoadhesion. The following three mucosal nanosystems are possible:

(a) Conventional or non-mucoadhesive nanosystems, which include nanoparticulate systems prepared without consideration of an interfacial interaction with the mucosa. In general, they have a superficial relationship with the mucosa without prolonging drug residence or promoting permeation across the mucus gel layer.

(b) Mucoahesive nanosystems, in which nanoparticles are formulated including a polymer with well-known mucoadhesive properties (e.g., poly(acrylic acid) or carbomers, alginate, chitosan, carrageenan, cellulose derivatives—methylcellulose, hydroxypropyl methylcellulose, carboximethylcellulose) which forms a coating capable to interact with the mucus layer (interpenetration), thus increasing the residence time of the system.

(c) Mucus-penetrating nanosystems, in which the coating layer includes a penetrating polymer, generally PEG or derivatives (e.g., poloxamers), which can promote the penetration of the nanoparticles to deeper regions of the mucus gel layer of the mucosa. A schematic representation of these nanosystems is shown in Figure 1.

In the case of vaginal disorders such as genital infections, hormone replacement therapy, vaginal dryness, etc. this classification can help the formulator to initiate nanoparticle development and to select the best type for a specific therapy [2,4].

Among reports in the literature, there is an increasing interest to develop nanoparticles of natural sources in the search for highly biocompatible and environmentally friendly materials.

## 4. Nanoparticles from Natural Polymers for Vaginal Treatments

**Figure 2:**
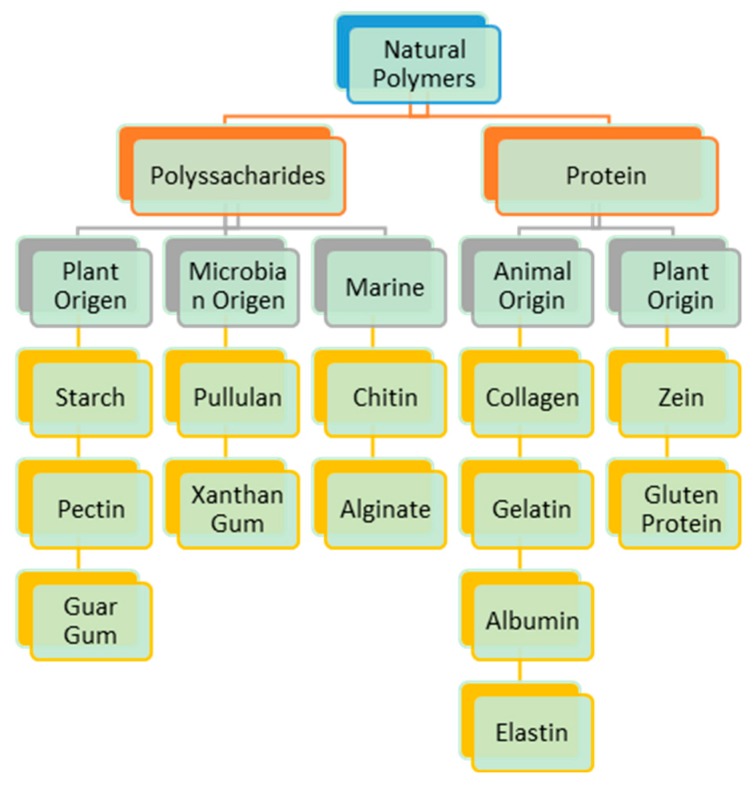
Classification of polymers of natural origin used to prepare nanoparticles with mucoadhesion characteristics for the vaginal route.

Natural polymers are materials of natural origin with pharmaceutically acceptable properties such as biocompatibility, low cost, raw material for controlled release platforms, availability, and lack of toxicity. Many of these polymers are obtained from plants, such as polysaccharides, while others are proteins such as zein, glutein, etc. These macromolecules are widely used in pharmaceutical formulations and play a significant role in the design of pharmaceutical dosage forms, particularly for controlled drug-release systems. However, and despite their already studied application in pharmaceutics for the vaginal route, there is little information regarding their use in the preparation of polymeric nanoparticles for vaginal systems [22,23,24]. Some research groups have reported the use of polysaccharides in polymeric nanoparticles that include those from algae, such as alginate, and others obtained by biotechnological processes. Figure 2 presents some of the natural polymers considered in the development of natural polymeric nanoparticles with potential use in vaginal mucosa.

In Figure 2, it is possible to highlight two polysaccharides—alginate and chitosan—which, in addition to being considered drug carriers, can control release in vaginal mucosa. They possess high antimicrobial activity, including their mucoadhesive properties. Polysaccharides exhibit increasing antioxidant properties, in particular chitosan, which also has the ability to enhance fibroblast proliferation in vitro [25,26]. Chitosan and alginates are the polysaccharides most reported in the preparation of nanoparticles for vaginal drug-delivery formulations. Their use is focused on vaccine, anti-conceptive, and microbicide delivery for the prevention or treatment of sexually transmitted diseases, as well as on cervical cancer [4,27]. A current tendency is to prepare nanoparticles combining natural polymers with natural drugs such as curcumin and piperine, which have recognized properties such as antioxidant, anti-inflammatory, antimicrobial, and anticancer activities [28]. Chitosan has the capability to interact with the mucus layer, facilitating mucoadhesion and consequently increasing drug-residence time at the mucosal surface, enhancing penetration into vaginal tissue cells [29].

Starch nanoparticles have been used to load neutrophil elastase inhibitor (ER 143) for anti-inflammatory testing in skin [2]. These authors showed that starch nanocapsules prepared by emulsion-solvent evaporation increase the drug permeation with maximal efficacy in the areas treated; thus, starch nanoparticles can be used for delivery drugs in mucosal tissues including the vaginal route.

Proteins are another type of polymer that have been considered for use in the development of drug-delivery nanoparticles for different administration routes, mainly owing to their characteristics such as low toxicity, stability, and ease of modification under different conditions (pH, ion exchange, etc.) [24]. Figure 2 shows other proteins of animal origin tested in drug delivery in different forms. Among these forms we find β-casein nanoparticles. These can be employed by chromotherapeutic oral-delivery drug systems and can be considered for mucosal delivery [30]. β-lactoglobulin has been employed as a lactoferrin encapsulant in microcapsules [31]. Zein, a protein of plant origin, has been used in combination with chitosan to prepare curcumin nanocapsules by the electrospray method with particle sizes ranging from 337–600 nm. Curcumin possesses anti-inflammatory, antimicrobial, and anticancer properties [28] and zein has exhibited bioadhesivity, biocompatibility, and the ability to penetrate mucus layers. However, curcumin shows negligible potential for systemic absorption when applied to vaginal mucosa. The prerequisite for successful topical vaginal therapy is the prolonged residence time of the drug; this effect is shown when the curcumin is trapped in liposomes, so the development of nanocapsules trapping these components represents an alternative to control the release of the drug, increasing the residence time [32].

Table 1 summarizes some representative examples of natural polymers used for the preparation of nanoparticles in the treatment of vaginal diseases, as well as loaded drugs, methods for their preparation, and the evaluation of their effectiveness in vivo or ex vivo. It should be noted that the majority of applications in vaginal mucosa are performed with chitosan as a polymer wall, due to its antimicrobial properties. These properties contribute to the control of systems employed in different treatments in the vaginal mucosa, implying the decrease of risk of contamination and dismissal of the effects of drug delivery [33]. Several mechanisms are reported in the literature to explain chitosan activity on wound healing. Chito-oligomers work as bricks in the synthesis of hyaluronan, which in turn promotes cell motility, adhesion, and proliferation, thus playing an important role in tissue repair [25].

The use of natural polymers for the development of drug release in vaginal mucosa has potential, as natural polymers present good bioadhesion capacity, biocompatibility, and low toxicity, and can be prepared easily. Natural polymers represent a strategy of use as polymers for the formation of nanoparticles. Additionally, they give rise to great interest because, due to their characteristics, they can easily interact with the vaginal mucosa, be internalized, and thus be useful as a strategy for drug administration and release.

In this regard, there is also a significant amount of information in the literature on this route of administration corresponding to the use of synthetic and biodegradable polymers for nanoparticle production. This has the advantage of having greater tools and knowledge of the interaction at the cellular level in in vitro models, and in aspects of toxicity, traceability, and efficacy in in vivo models.

## 5. Synthetic and Biodegradable Polymer-Based Nanoparticles

Polymeric nanoparticles are formulated using natural or synthetic polymers, adhesive or non-bioadhesive, with a high level of biocompatibility [6]. Biocompatible and biodegradable polymers approved for human use by the U.S. Food and Drug Administration (FDA) are preferably used to produce nanoparticles, e.g., poly(DL-lactic acid), poly(lactic-co-glycolic acid), polycaprolactone, polyacrylates, polymethacrylates, cellulose derivatives, triblock copolymers of poly(ethylene oxide)/poly(propylene oxide) or poloxamers), poly(vinyl alcohol), poly(ethylene glycol), and alginate [2]. Some of the most significant synthetic and biodegradable polymers are those based on lactic and glycolic acids. The polyester polylactic-co-glycolic acid (PLGA) has excellent reproducible mechanical and physical properties, controllable release, and predictable degradation kinetics; likewise, its extensive safety, biocompatibility, and biodegradability have been recorded. PLGA is acceptable for delivering drugs via all major routes of administration, including the vaginal route. This polymer is one of those most employed for investigations focusing on the delivery of antiretroviral drugs and small interfering RNA (siRNA) for Human Immunodeficiency Virus (HIV) prophylaxis, as well as microbicides [5,38]. Table 2 presents some examples of polymer nanoparticles designed for vaginal drug delivery.

For the development of polymeric nanoparticles for vaginal drug delivery, it is important to focus on vaginal mucus as a barrier and a clearance mechanism that can limit vaginal retention [1,4]. Phenomena such as genital distribution, mucosal retention, and cell/tissue uptake are dependent not only on the vaginal environment, but also on the inherent properties of nanoparticles, namely composition, size, and surface chemistry [2]. Considering the mechanisms of natural cleaning of the vagina that give rise to poor mucous retention, the use of mucoadhesive vaginal drug-delivery systems could be highly recommendable because they extend cervicovaginal residence. Nevertheless, adhesive interactions between nanoparticles and mucin exhibit some disadvantages, for example the following: (i) they disrupt the natural mucus barrier; (ii) they limit nanoparticle diffusion, and thus are mainly established at the top layers of mucus; and (iii) they impair the vaginal distribution of nanoparticles and their ability to effectively cross the CVM and deliver the drug payload to the underlying mucosal surface [2,51]. Two of the most commonly used strategies to increase the mucoadhesion of nanosystems are as follows: (i) providing specific surface chemical properties that interact with the negatively charged mucin; and (ii) bulk preparation or surface modification of nanoparticles with mucoadhesive polymers [51]. On the other hand, mucus-penetrating particles can rapidly penetrate the vaginal mucus, improving vaginal drug distribution and retention, rather than being trapped and more rapidly cleared in the outer, luminal layers of the mucus [1].

Polyethylene glycol (PEG) is the most widely used “stealth” polymer in the drug-delivery field [52]. PEG has been widely used as a coating for nanoparticles and biomaterials to resist the non-specific adsorption of biomolecules, particularly serum proteins, and to enhance the mucosal penetration of PLGA nanoparticles [53]. It has also been reported that polystyrene (PS) nanoparticles coated with low molecular weight (2 or 5 kDa) PEG diffuses rapidly in human CVM, but PS-PEG nanoparticles coated with 10 kDa PEG were immobilized [52]. Lai and coworkers [54] used high-speed multiple-particle tracking to quantify the transport rates of individual polymeric particles of various sizes and surface chemistries in samples of fresh human CVM. These authors confirmed that carboxylate-modified PS nanoparticles with a size of 100–500 nm were completely immobilized in human CVM. In the meanwhile, the same particles coated with PEG diffused through CVM with rates up to one fourth as fast as they would be diffused in pure water. In addition, large (200–500 nm) particles diffused much more rapidly than 100-nm particles in human CVM, for both COOH- and PEG-modified surfaces. Surprisingly, this research group reported that nanoparticles larger than the previously reported CVM mesh spacing (~20–200 nm) are capable of rapid transport in CVM if they are coated with the mucoresistant polymer, low-molecular-weight PEG. This result suggested that (i) mucus is a mesh of proteins with low viscosity fluid-filled pores larger than 500 nm where nanoparticles 100–500 nm in size diffuse throughout (if non-adhesive); and (ii) nanoparticles without PEG coatings become entrapped in mucus by adhesive interactions [52,54].

Some years later, it was confirmed that nanoparticles modified with PEG penetrate through mucus, improving drug delivery in the cervicovaginal tract. Cu and co-workers [55] explored the fate of PLGA nanoparticles in the mouse reproductive tract after intravaginal delivery. Negatively charged PLGA nanoparticles, and PLGA nanoparticles modified with Avidin (Avid-NP) or 2 kDa PEG (PEG-NP), were prepared by the emulsion evaporation method. Coumarin-6 (C6) was used as a fluorescent marker. Results showed that unmodified PLGA particles exhibited significantly lower retention in the lumen after intravaginal delivery than particles modified with Avidin or PEG because the surface properties of nanoparticles influence their retention and transport in mucus. PEG nanoparticles, which have a neutral surface, can penetrate the mucosal epithelium more efficiently, and mucoadhesive Avid-NP do not undergo leakage to the same extent as unmodified PLGA nanoparticles. It is important to remark that the porcine vaginal mucosa and the human vaginal mucosa consisted of stratified squamous epithelium supported by connective tissue and of the lamina propria, and they have a similar lipid composition, important for barrier function [44,56].

Recently, Ariza-Sáenz et al. [44] prepared PLGA nanoparticles covered with glycol-chitosan and loaded with an HIV fusion inhibitor peptide (E2) and evaluated their ex vivo penetration in vaginal mucosa and their in vivo penetration rate in female swine. Nanoparticles with E2 (E2-NP) and 5(6)-carboxyfluorescein (E2-FAM-NP) as well as empty nanoparticles were obtained by a modified double-emulsion method and were covered with chitosan conjugated with ethylene glycol. Mean sizes were 305 and 326 nm for E2-NP and E2-FAM-NP, respectively. Encapsulation efficiency for the peptide was greater than 62% in both cases. Assays with cryopreserved mucosae were conducted ex vivo using Franz cells and confocal microscopy to evaluate the transit of the E2-FAM-NP to the vaginal epithelium. The physicochemical and morphological features of E2-NP enable their mobility across the mucus, reaching the epithelium, and spreading within the adjacent epithelial layers. In addition, if peptide E2 is released, it may penetrate the mucosa through lipid bilayers and interact with cellular membranes, increasing their concentration near the fusion sites within the intercellular space. E2-NP constitutes a new alternative to inhibit HIV infection.

Even though PEG has dominated the field of surface coatings, Deng and coworkers [53] proposed a new method that improves the stealth, aggregation-resisting, mucus-penetrating, and local retention properties of PLA nanoparticles, without the use of PEG. The authors synthesized a copolymer of PLA and hyperbranched polyglycerols (HPG) by a one-step esterification, and nanoparticles were produced from a single emulsion using PLA-HPG. Subsequently, the same research group [45] proposed the intravaginal use of bioadhesive nanoparticles prepared with poly(lactic acid)-hyperbranched polyglycerols (PLA-HPG) and loaded with the antiretroviral elvitegravir (EVG). This method involves the conjugation of poly(lactic acid) (PLA) to hyperbranched polyglycerols (HPG) to produce PLA-HPG nanoparticles, with the HPG forming a corona on the nanoparticle surface that provides stealth properties to the nanoparticles and avoids adhesion to proteins and protein-rich surfaces. When the surface coating of these nanoparticles is oxidized by simple exposure to sodium periodate, the vicinal diols on HPG are converted into aldehydes capable of forming stable Schift base interactions with amine group-enhanced bioadhesion. Non-bioadhesive and bioadhesive PLA-HPG nanoparticles loaded with EVG were prepared (EVG-PLA-HPG-NP and EVG-PLA-HPG-BNP, respectively) and characterized. The distribution and retention of bioadhesive and non-bioadhesive nanoparticles loaded with a fluorescent dye in the reproductive tract after vaginal administration in mice was analyzed. In vitro release studies using dialysis tubes showed that after an initial burst release of EVG (~40%) within the first 8 h, both EVG-PLA-HPG-NP and EVG-PLA-HPG-BNP demonstrated slower and more stable release up to 3 days in the simulated vaginal fluid (SVF). EVG-PLA-HPG-BNP distribution was widespread through the reproductive tract and retained along the vaginal epithelium at a rate nearly five times higher than EVG-PLA-HPG-NP after 24 h.

A challenge in formulating drug-loaded mucus-penetrating particles lies in the adequate selection of the surfactant, because commonly used surfactants either yield particles that adhere to mucins, or particles that do not provide efficient drug encapsulation. For example, PVA is an uncharged and relatively hydrophilic polymer very frequently employed as a surfactant during particle formulation by nanoprecipitation or emulsion methods that provide high drug-loading efficiencies but that generate mucoadhesive nanoparticles [43,57]. The effect of PVA coating on the transport of nanoparticles in fresh human CVM has been extensively studied by the research group of J. Hanes [57] using multiple particle tracking, a technique that allows quantitative measurements of the motions of hundreds of individual particles. These authors analyzed nanoparticles prepared with different polymers such as polystyrene, PLGA, and diblock copolymers of PEG-PLGA. The results suggested that conventionally formulated PVA-coated particles display mucoadhesive properties and are immobilized in CVM regardless of PVA molecular weight (2–25 kDa), PVA concentration (0.01–1%), or particle core materials. The results showed that PVA-coated PLGA nanoparticles 270 nm in diameter were immobilized by the mucus due to surface adsorption, although their sizes were comparable to that of mucus-penetrating PS-PEG nanoparticles (232 nm).

In a later study, Popov and collaborators [58] evaluated nanoparticle transport in human CVM ex vivo as measured by multiple particle tracking and bulk permeation methods. These authors demonstrated that nanoparticles coated with PVA could be able to penetrate mucus or to act as mucoadhesive particles. Nanoparticle behavior depends on the degree of PVA hydrolysis. In summary, nanoparticles coated with PVA that are ≥95% hydrolyzed were immobilized in mucus; however, nanoparticles coated with PVA that have hydrolysis degrees of <95%, and at least as low as 75%, can aid particle mobility in mucus.

A remarkable strategy for obtaining biodegradable nanoparticles that easily penetrate the human CVM was introduced by Mert et al. [43]. This strategy involves the use of a novel surfactant molecule composed of activated vitamin E succinate conjugated to 5 kDa PEG (VP5k). Nanoparticles were prepared by a solvent diffusion/nanoprecipitation technique and PLGA was fluorescently tagged with doxorubicin in order to visualize particle motions in cervicovaginal mucus by video microscopy. Results demonstrated that VP5k-coated PLGA nanoparticles rapidly penetrate human cervicovaginal mucus, whereas PLGA nanoparticles coated with polyvinyl alcohol or vitamin E conjugated to 1 kDa PEG were trapped. Moreover, a high loading of a small molecule drug (Paclitaxel) was encapsulated in VP5k-coated PLGA nanoparticles. In vitro-release kinetic studies in phosphate-buffered saline (PBS) solution pH 7.4 (37 °C) of these nanoparticles exhibited minimal burst effect, followed by paclitaxel sustained release for ~5 days.

Recently, Frank and collaborators [46] proposed a combined strategy to increase the adhesion and penetration of drug though the vaginal mucosa. This proposal involves the development of polymeric nanocarriers, explicitly nanocapsules, and the subsequent incorporation of them into a mucoadhesive semisolid formulation. Two different formulations were studied: chitosan-coated Imiquimod-loaded poly(ε-caprolactone) nanocapsules incorporated into hydroxyethylcellulose gel, and Imiquimod-loaded poly(ε-caprolactone) nanocapsules incorporated into chitosan hydrogel. Nanocapsules were produced by the interfacial deposition of a polymer. Nanocapsules coated with a cationic polymeric (chitosan) did not increase adhesivity to porcine vaginal mucosa within the period of the experiment by means of a tensile stress tester. In contrast, there was a significant difference between the mucoadhesivity of hydrogels; chitosan hydrogel with chitosan-coated nanocapsules presented higher mucoadhesion than chitosan hydrogel with uncoated nanocapsules. In addition, the authors proposed an innovative method employing radar plots to compare the proposed formulations in terms of drug mucoadhesivity by interaction, permeation, and the Imiquimod retained. After the global analysis, the formulation based on chitosan gel and Imiquimod-loaded polymeric nanocapsules demonstrated the most promising performance for Human Papillomavirus Infection (HPV) treatment.

In our research group, PLGA nanoparticles were recently developed with chitosan surface modification in order to control vaginal clotrimazole delivery for the treatment of vaginal infections generated by *Candida albicans* [42]. Microbiological studies indicated that clotrimazole (at a level of 10 µg/mL) increased its effectiveness against *C. albicans* by four-fold when incorporated into PLGA-chitosan nanoparticles.

The use of biodegradable polymers is attractive; however, there is also significant evidence in the literature of improvements in pharmacological properties using non-biodegradable polymers, but ones that are highly biocompatible, safe, and possess practically no toxic effects.

## 6. Nanoparticles Prepared from Synthetic Non-Biodegradable Polymers

As an alternative option to polymers of natural origin, we find the use of synthetic polymers, which generally represents higher control in physicochemical properties [59,60]. Therefore, some non-biodegradable, but biocompatible synthetic polymers have been employed as carriers of drugs for different vaginal conditions. One of the polymers of this type with broad applications is the Eudragit^®^ RS 100 or poly(ethyl acrylate-co-methyl methacrylate-co-trimethylammonioethyl methacrylate chloride). Eudragit^®^ RS 100 is a copolymer of ethyl acrylate, methyl methacrylate, and a low content of methacrylic acid ester with quaternary ammonium groups. This polymer is soluble in organic solvents, which facilitates its incorporation in the majority of traditional methodologies for obtaining nanoparticulate carrier systems. This polymer employed for targeted drug release has been also used for vaginal diseases in the form of nanospheres and nanocapsules (as the polymer surrounding a liquid). In the search for new oil cores with antifungal properties, there has been increasing interest in the preparation of polymeric nanocapsules with vegetable oils; for example, coconut oil has high nutritional and pharmaceutical value [61]. In terms of composition, it has a lower amount of unsaturated fatty acids than others. In addition, coconut oil has antifungal and antioxidant activities. With this background, coconut oil-core nanocapsules from Eudragit^®^ RS 100 were proposed for clotrimazole delivery intended to improve solubility and to reduce mucosal irritation in the treatment of vulvovaginitis caused by *C. albicans* [62]. Preliminary assays demonstrated that coconut oil proved to be a suitable raw material for the preparation of nanocapsules of Eudragit^®^ RS 100, since the oil did not produce its dissolution.

Nanocapsule preparation was performed through the interfacial deposition of Eudragit^®^ RS 100. With the aim of forming an emulsion, the organic phase constituted of polymer, acetone, Span^®^ 80, clotrimazole, and coconut oil was poured into an aqueous dispersion of Tween^®^ 80 under magnetic stirring. After this, organic solvent was removed by evaporation under reduced pressure. Final clotrimazole concentrations of 1.0 and 3.0 mg/mL were obtained [61]. The final formulation was characterized as homogeneous and exhibited a milky appearance characteristic of colloidal systems, with a mean particle size lower than 200 nm and a polydispersity index lower than 0.2. A positive zeta potential was related to the quaternary ammonium groups of the polymer, facilitating attraction to the negatively charged polymer on the mucosal surface and increasing residence time. Interestingly, an encapsulation efficiency of 99.9% was reported with a controlled drug release of less than 40% in 8 h, and with high antifungal activity found in both coconut oil and clotrimazole [61]. In another study, the same research group compared nanocapsules with another formulation and with the same polymer composition but with a core of medium-chain triglycerides, and its antifungal activity was lower than that of the coconut oil-core [63]. Recently, the same researchers developed a similar formulation of a mucoadhesive gel of pemulen/pullulan containing clotrimazole-loaded cationic nanocapsules, demonstrating adequate mucoadhesion and vaginal permeation with Eudragit^®^ RS 100 and medium-chain triglycerides for the oil-core [64]. The usual method consisted of the solubilization of Eudragit^®^ RS 100, Span^®^ 80, and medium-chain triglycerides in acetone; subsequently, this was injected into an aqueous dispersion of Tween^®^ 80 with magnetic stirring. Afterward, the solvent was removed by evaporation under reduced pressure, and the concentration was adjusted [61], namely, by means of the interfacial deposition of pre-formed polymer method. Another study confirming the ability of Eudragit^®^ RS 100 to form nanocapsules comprises the work of the same research group on the development of rose-hip-oil-loaded nanocapsules. The developed formulation showed particles of 158 nm, with a potential zeta of 9.8 mV, and prevented the oxidation of the oil. Finally, the formulation was reported as suitable for cutaneous use [65]. With a special focus on the control of HIV, principally for HIV infections during pregnancy, the first-line drug in the treatment of prophylaxis of HIV-1 infections, efavirenz, was encapsulated in nanospheres of Eudragit^®^ E100 for controlled drug release and applied in aerosol with good stability in the foam and with fine bubbles, allowing a possible high level of spreading and high contact time. Nanospheres were obtained by the emulsion-solvent evaporation method employing Pluronic^®^ F68 and sodium alginate as stabilizers [66]. Other studies confirmed its in vivo efficacy [67]. With the same aim, recently, the use of Eudragit^®^ L 100 was reported to produce polymeric nanoparticles containing disopropyl fumarate and emtricitabine, two antiretroviral drugs, by means of a semi-automated spray-drying method. The nanoparticles were designed to be used as a prophylactic treatment against vaginal HIV transmission. Subsequently, the formulation was incorporated into PVA films and/or pectin to achieve a more controlled release. Burst release was decreased and a slow release profile was obtained up to 8 h [68]. Other works have involved the design of carriers to confer stability against acidic and enzymatic environments by pH-sensitive nanoparticles of Eudragit^®^ S-100. This system was obtained with fluorescent markers by the modified quasi-emulsion solvent-diffusion method. Eudragit^®^ S-100 nanoparticles were taken up by the vaginal cells and rapidly released at physiological pH. No cytotoxicity was reported in vaginal cell lines and this could be a potential carrier for vaginal delivery, avoiding the lack of stability [69].

The effectiveness of Eudragit^®^ RS 100 for vaginal treatments has been confirmed with traceability studies using Nile red as a model of lipophilic substance in the vaginal route of administration, by means of the interfacial deposition of pre-formed polymer method and Miglyol^®^ 812 as the oil-core. The main advantage of this type of polymer is the positive charge that increases the capacity of mucoadhesion, residence time for prolonged release, and, above all, increased penetration capacity in the vaginal mucosa, unlike other nanocapsules, regardless of the gel in which it is incorporated. The formulation reached a depth of up to 3800 µm in the vaginal mucosa [70].

Toxicity studies of Eudragit^®^ RS 100 nanocapsules on primary lines of fibroblasts and keratinocytes have not, to our knowledge, exhibited significant changes and irritation. The main effects, as in most submicron drug transporters, are attributed to surface active agents [71].

In this regard, the modification of the surface and the importance of the vehicles employed for the administration of the nanoparticles are evidenced.

## 7. Vehicles for the Administration of Polymeric Nanoparticles by the Vaginal Route

Although nanoparticle suspensions sometimes have been directly administered into the vaginal cavity, aspects such as retention, bioadhesion, and convenience are not complete satisfied. The use of vehicles in which polymeric nanoparticles are included is another issue to consider in the development of vaginal drug-delivery systems based on polymeric nanoparticles. In principle, nanoparticles should be maintained unaltered once they are included in the vehicle formulation. They also should be well dispersed and distributed in the vehicle, and the vehicle should contribute to rapid diffusion through the vaginal mucus, preferably minimizing mucin-nanoparticle adhesive interactions. On the other hand, formulation should address several considerations, such as ease of application, bioadhesivity, biocompatibility, optimal retention time, rheological behavior, maintenance of optimal pH, osmolality, promotion of absorption of the nanoparticles or drug, chemical stability of the drug, modulation of drug release, and integrity of the nanoparticles, among others.

Hydrogels are the most popular dosage form for the vaginal administration of drugs, and these have been extensively employed as vehicles for the incorporation of nanoparticles in formulations intended for the treatment of vaginal diseases or for transmucosal drug delivery through the vagina. In fact, VivaGel^®^, a vaginal microbicide gel that includes the dendrimeric active SPL7013 in a carbomer base, is one of the successful commercial examples of the potential of hydrogels in vaginal application.

A type of hydrogel, the thermosensitive gel, is an attractive dosage form because these gels can be administered in the liquid state; subsequently, gelation occurs at body temperature in the vaginal cavity. Poloxamer (407 and 188), a triblock copolymer surfactant, is one of the polymeric materials employed for the formulation of thermosensitive hydrogels. Date et al. [72] developed a thermosensitive vaginal gel that includes nanoparticles of raltegravir/efavirenz; both Poloxamers 407 and 188 were utilized as temperature-sensitive gelators. Peng et al. [73] reported the inclusion of nanoparticle plasmids coupled with the C32-117 poly(β-amino ester) polymer, which was used to target DNA-nanoparticle uptake in cervical pre-neoplastic cells. Ramyadevi et al. [39,74] utilized Poloxamer 407 to prepare in situ thermosensitive gel systems; the authors reported the incorporation of Acyclovir loaded into polymeric nanoparticles composed of ethyl cellulose/PVP/Eudragit^®^ RS blends, and the incorporation of the nanoparticles was achieved in a dry state. Sometimes nanoparticles could be incorporated from aqueous suspensions; however, high concentrations of solids in the suspension may be necessary to comply with the drug dose or to reduce the effect of the dilution of the gel due to the addition of water. Another issue to consider is the optimal thermogelation point of the gel; this requires being set between 30 and 33 °C to maintain the thermosensitive gels in a liquid state even in sub-tropical and tropical countries. At the same time, the thermogelation temperature should not be too near 37 °C [72]. Additional important issue in the formulation of hydrogels for vaginal application is the pH: values should fall preferably between 3.8 and 4.5, and buffer systems based on citrate salts are commonly used in the formulation of vehicles. It is noteworthy that the gelling agent should preferably be non-pH-sensitive.

In recent years, chitosan-based gels are another type of hydrogel that has attracted attention for vaginal applications. Chitosan is nontoxic, biocompatible, biodegradable, possesses good mechanical properties, good film-forming ability, and is bioadhesive; also, fungicidal and antimicrobial properties have been found. Frank et al. [70] found that gels with a chitosan concentration of 2.5% *w*/*w* were those most suitable for vaginal delivery. Chitosan hydrogels exhibited higher adhesion to vaginal mucosa; in addition, the study indirectly revealed low interaction between the vehicle and the fluorescent-marked Eudragit^®^ RS nanoparticles because penetration of the lipophilic molecule model into the mucosa was high in comparison to when this was not nanoencapsulated.

Cellulose-based hydrogels have been also developed as vehicles for the administration of nanoparticles in the vagina. Frank et al. [46] reported the preparation of Imiquimoid-loaded nanocapsules dispersed in water and incorporated in hydroxyethylcellulose gel to compare chitosan hydrogels containing nanocapsules. In this study, it was shown that no particle agglomeration or aggregation occurred during the process of hydrogel production by the analysis of particle diameter before and after incorporation into the gel. Yang et al. (2013) also reported the use of hydroxyethylcellulose; in their study, saquinavir-loaded nanoparticles composed of PLGA were resuspended in PBS (pH 5.0) and loaded into hydroxyethylcellulose gel. The homogeneity of nanoparticles in the gel was confirmed by random sampling of different parts of the gel, and then fluorescence was measured.

Another system proposed by de Lima et al. [64] used a combination of a polymer composed of acrylic block copolymers (Pemulen^®^ TR1) and pullulan, a water-soluble, biodegradable, and biocompatible polysaccharide. Clotrimazole-loaded nanocapsules composed of Eudragit^®^ RS were also included in the hydrogel. The content of the drug in the nanocapsules was not altered by the vehicle during the 60 days, and the particle size did not change.

Not only hydrogels have been proposed as vehicle for the incorporation of polymeric nanoparticles in vaginal formulations; polymeric solid dosage forms have likewise been investigated. Vaginal films are promising systems as they possess some advantages over hydrogels, in addition to their technological advantages such as reproducibility or the possibility of incorporating drug-loaded nanoparticles immediately prior to the casting and drying of films. Moreover, when vaginal films are administered, no introduction of extra fluids is required, implying no product leakage upon use. Additionally, this solid dosage form can be used to stabilize drugs that are susceptible to degradation in aqueous environments [75,76].

Gu et al. [77] reported the preparation of siRNA nanoparticles enhanced with anti-HLA-DR antibody incorporated into a polymeric film composed of PVA and λ-carrageenin. The film disintegrated in 10 min, and the complete release of nanoparticles was evaluated. Machado et al. [38] described the preparation and evaluation of PVA and HPMC film that included tenofovir-loaded, PLGA-based nanoparticles. Glycerin at 10% was used as a plasticizer. Commonly, a dual in vitro drug-release profile is observed for films: the first free drug is immediately released to form the film surface, then sustained release is mediated by the nanoparticles. Cunha-Reis et al. [40] also reported the incorporation of efavirenz-loaded PLGA nanoparticles in films composed of 72% HPMC, 18% PVA, and 10% glycerin. Recently, Pereira et al. [68] prepared double-layered films containing the antiretroviral drugs tenofovir disoproxil fumarate and emtricitabine loaded in Eudragit^®^ L 100 nanoparticles. First, the nanoparticles were obtained by nano-spray-drying, then dispersed on top of plain film, and finally covered by an additional layer of plain film before binding the system using a hydraulic press. The films were formed by the solving casting technique using PVA and pectin as film-forming agents, and PEG 4600/glycerin (1:1 *w*/*w*) as plasticizers.

Finally, Rossi et al. [25] explored the incorporation of amoxicillin-loaded chitosan nanoparticles in fast dissolving matrices composed of PVP, mannitol, and glycine. The matrices were obtained by freeze-drying from aqueous stock solutions of PVP 3% *w*/*w*, mannitol 10% *w*/*w*, and glycine 5% *w*/*w*. The rapid release of nanoparticles in simulated vaginal fluid was characterized by unchanged size.

## 8. Conclusions and Future Trends

The physiology of the vagina as a drug-administration site and the constant changes in its composition represent a challenge for the optimal development of pharmaceutical formulations. Controlled-release nanoparticles can reduce bacterial and fungi tolerance and decrease the frequency of administration due to the prolonged release of highly interpenetrated particles in the vaginal tissue, in an enhanced activity of the drug, polymer, oil-core and, in some cases, also of the administration vehicle (hydrogel). For this purpose, hydrogels can demonstrate other functionalities, such as thermogelation, in addition to good mechanical properties, film-forming ability, and bioadhesion. It is important to highlight the multifunctionality of several natural components for the preparation of nanoparticles, which reduces the possibility of multi-drug therapy. The majority of classical methods of nanoparticle preparation from synthetic pre-formed polymers or from polymers of natural origin are in accordance with the requirements for obtaining nanoparticles for vaginal administration with minor modifications. They are fast, reproducible, and possess a high concentration in solids. Future trends could suggest a special focus on the modification of the nanoparticle surface to increase the pharmacological effect. This conjugation involves maintaining stability, and adsorbing or crosslinking new polymers. Some issues of concern, such as traceability, safety, bioethical issues, toxicity hazards, and physiological and pharmaceutical challenges, are yet to be resolved by scientists. Finally, polymer-based nanoparticles comprise a viable alternative to counteract different pathologies associated with the anatomical region of the vagina, but interestingly, also as a tool for prophylaxis.

## Figures and Tables

**Table 1:** Application of natural polymers in nanoparticles preparation for vaginal mucosa.

Loaded Drug	Natural Polymer	Preparation Method	Treatment	Main	Findings
Afamoxicillin trihydrate	Chitosan ascorbate	Ionotropic gelation	Atrophic Vaginitis	Particle size was 210–268 nm according to the % of Afamoxicillin encapsulated	Antibacterial expressed as minimal concentration of antibiotic was 0.004% for *S. pyogenes* and 0.0001% for *E. hirae* [25]
Ascorbic acid	Chitosan	Ionotropic gelation	Cervical Cancer	Particle size decreased with increase of ascorbic acid load in chitosan	An increase in chitosan-ascorbic acid concentration declined the survival of HeLa cells [34]
Insulin	Chitosan	Ionotropic gelation	Model for peptides delivery	Cylindrical sponges prepared by freeze-drying with different excipient type (mannitol, sucrose, gelatin)	Confirmed the good penetration properties of nanoparticles for use in the treatment of sexually transmitted diseases [29]
Silver saccharinate (AgS)	Alginate	Reverse emulsification	HSV-2 and *Neisseria gonorrhoeae* inhibition	Silver saccharinate showed excellent stability in vaginal fluid simulant	Silver saccharinate showed prevention both of HIV-1 infection and STIs via sexual intercourse in women [35]
Tenofovir	Gelatin	Desolvation method	Sexual transmission of HIV in women	Particle size was 294.7–445 nm	The in vitro dissolution study for nanoparticle formulation revealed sustained release of Tenofovir over 8 h [36]
Ciprofloxacin Riboflavin	Ovomucin	Nano-precipitation	Not reported	Ovomucin is suitable for encapsulating heat-sensitive drugs	Ovomucin particles exhibited sustained release; particles were resistant to degradation in both simulated mucus media and intestinal fluid [37]

HSV-2: herpes simplex virus type 2; STIs: sexually transmitted infections; HIV-1: human immunodeficiency virus.

**Table 2:** Examples of synthetic and biodegradable polymer-based nanoparticles for vaginal drug delivery.

Drug or Active Compound	Polymer(s)	Preparation Method	Diameter (nm)	AE, EE, or DL (%)	Findings
Tenofovir	PLGA 50:50	Double emulsion-solvent evaporation	118	AE = 18.5	When 12.5% of PLGA was substituted for stearylamine, the drug association increased to 48.4%. Nanoparticles were incorporated into a HPMC/PVA-based film [38].
Acyclovir	PVPK30-EC (F1)	Nano-precipitation	403 (F1)	DL = 80	Freeze-dried nanoparticles were incorporated into Pluronic^®^ F-127 gel [39].
	PVPK30-ERSPO (F2)		99 (F2)		
Efavirenz	PLGA 50:50	Emulsion-solvent evaporation	275	AE = 96.8	Efavirenz-loaded nanoparticles and free tenofovir were incorporated into fast-dissolving films (72% HPMC, 18% PVA, 10% glycerin) [40].
Dapivirine	PLGA 50:50	Emulsion-solvent evaporation	168	DL = 1.8	An enhanced safety profile of drug-loaded nanoparticles over free dapivirine. A decrease in drug permeability with increased epithelial cell membrane retention [41].
				AE = 90.2	
Clotrimazole	PLGA 50:50	Emulsification-diffusion	492	DL = 68.2	Mucoadhesive chitosan-coated nanoparticles showed an increment in antifungal activity inhibition compared to drug solution [42].
Paclitaxel	PLGA 50:50	Solvent diffusion/nano-precipitation	245	DL = 7.9	Mucus-penetrating nanoparticles. In vitro paclitaxel sustained release for 5 days [43].
An HIV fusion inhibitor peptide (E2)	PLGA 50:50	Double emulsion method-solvent evaporation	305	EE = 62.0	Mucoadhesive nanoparticles covered with glycol-chitosan with mobility across the mucus [44].
Elvitegravir (EVG)	PLA conjugated with HPG	Nano-emulsion protocol	135 non-adhesive	EE = 94	Both nanoparticles demonstrated a slower and more stable release up to 3 days in simulated vaginal fluid [45].
			131 bioadhesive		
Imiquimod	PCL	Interfacial deposition	199 uncoating	EE = 97 uncoating	Chitosan-coated PCL nanocapsules were incorporated into HEC gel, and uncoated PCL nanocapsules were incorporated into chitosan hydrogel. The latter formulation showed the most promising performance for the treatment of human papillomavirus [46].
			213 coating with chitosan	EE = 57 coating with chitosan	
Dapivirine	PCL	Solvent displacement method	194 (PEO-PPO-PEO)	AE = 97.8, DL = 12.8 (PEO-PPO-PEO)	Three surface-engineered dapivirine-loaded, PCL-based nanoparticles were obtained using PEO-PPO-PEO, SLS, or CTAB as surface modifiers. Negatively charged nanoparticles were stable up to 1 year; as for CTAB–PCL nanoparticles, particle aggregation was observed [47].
			178 (SLS)	AE = 97.5, DL = 12.8 (SLS)	
			185 (CTAB)	AE = 97.8, DL = 12.8 (CTAB)	
siRNA against nectin-1	PLGA 50:50	Double emulsion-solvent evaporation	299 (3:1)	EE = 92 (3:1)	Three complexes were formed between the siRNA and spermidine at molar ratios of the polyamine nitrogen to the nucleotide phosphate (N:P ratio) of 3:1, 8:1, or 14:1, ahead of nanoparticles preparation. The intravaginal administration with nanoparticles of PLGA encapsulating siRNA molecules was effective for the prevention of genital HSV-2 infections in mice [48].
			331 (8:1)	EE = 82 (8:1)	
			323 (14:1)	EE = 43 (14:1)	
Dapivirine	PCL	Solvent displacement method	198 (PEO-PPO-PEO)	AE = 97.3, DL = 12.7 (PEO-PPO-PEO)	Nanoparticles with three different surface modifiers: PEO-PPO-PEO, SLS, or CTAB. Antiretroviral activity of nanoparticles was determined in different cell models, as well as their cytotoxicity. CTAB-PCL nanoparticles provided higher intracellular concentrations of dapivirine than the two other formulations in VK2/E6E7 human vaginal epithelial cells [49].
			182 (SLS)	AE = 97.6, DL = 12.7 (SLS)	
			193 (CTAB)	AE = 97.9, DL = 12.8 (CTAB)	
Dapivirine	PCL	Solvent displacement method	199	AE = 97.6	Dapivirine-loaded PCL nanoparticles coated with PEO-PPO-PEO. Nanoparticles were rapidly eliminated after vaginal administration (mouse model) but able to distribute throughout the vagina and lower uterus, and capable of tackling mucus and penetrating the epithelial lining [50].
				DL = 12.7	

AE: drug association efficiency; DL: drug loading; EE: entrapment efficiency; CPT: camptothecin; CTAB: cetyltrimethylammonium bromide; ERSPO: Eudragit^®^ RSPO; HEC: hydroxiethylcellulose; HPG: hyperbranched polyglycerols; HPMC: hydroxypropyl methylcellulose; PCL: poly(ε-caprolactone); PEO–PPO–PEO: triblock copolymer of poly(ethylene oxide) (PEO) and poly(propylene oxide) (PPO); PLA: poly(lactic acid); PLGA: poly(lactic-co-glycolic acid); PVA: poly(vinyl alcohol); PVPK30: polyvinyl pyrrolidone K30; siRNA: short interfering RNA molecules; SLS: sodium lauryl sulfate; F1: formulation 1; F2: formulation 2.
